# Transcriptome Analysis of Mango (*Mangifera indica* L.) Fruit Epidermal Peel to Identify Putative Cuticle-Associated Genes

**DOI:** 10.1038/srep46163

**Published:** 2017-04-20

**Authors:** Julio C. Tafolla-Arellano, Yi Zheng, Honghe Sun, Chen Jiao, Eliel Ruiz-May, Miguel A. Hernández-Oñate, Alberto González-León, Reginaldo Báez-Sañudo, Zhangjun Fei, David Domozych, Jocelyn K. C. Rose, Martín E. Tiznado-Hernández

**Affiliations:** 1 Coordinación de Tecnología de Alimentos de Origen Vegetal, Centro de Investigación en Alimentación y Desarrollo, A. C. Hermosillo, Sonora, México; 2 Plant Biology Section, School of Integrative Plant Science, Cornell University, Ithaca, NY, USA; 3 Boyce Thompson Institute, Cornell University, Ithaca, NY, USA; 4 Red de Estudios Moleculares Avanzados, Instituto de Ecología A. C., Cluster Biomimic^®^, C. Xalapa, Veracruz, México; 5 CONACYT-Coordinación de Tecnología de Alimentos de Origen Vegetal, Centro de Investigación en Alimentación y Desarrollo A.C. Hermosillo, Sonora, México; 6 US Department of Agriculture/Agriculture Research Service, Robert W. Holley Center for Agriculture and Health, Ithaca, NY, USA; 7 Department of Biology, Skidmore Microscopy Imaging Center, Skidmore College, Saratoga, Springs, NY, USA

## Abstract

Mango fruit (*Mangifera indica* L.) are highly perishable and have a limited shelf life, due to postharvest desiccation and senescence, which limits their global distribution. Recent studies of tomato fruit suggest that these traits are influenced by the expression of genes that are associated with cuticle metabolism. However, studies of these phenomena in mango fruit are limited by the lack of genome-scale data. In order to gain insight into the mango cuticle biogenesis and identify putative cuticle-associated genes, we analyzed the transcriptomes of peels from ripe and overripe mango fruit using RNA-Seq. Approximately 400 million reads were generated and *de novo* assembled into 107,744 unigenes, with a mean length of 1,717 bp and with this information an online Mango RNA-Seq Database (http://bioinfo.bti.cornell.edu/cgi-bin/mango/index.cgi) which is a valuable genomic resource for molecular research into the biology of mango fruit was created. RNA-Seq analysis suggested that the pathway leading to biosynthesis of the cuticle component, cutin, is up-regulated during overripening. This data was supported by analysis of the expression of several putative cuticle-associated genes and by gravimetric and microscopic studies of cuticle deposition, revealing a complex continuous pattern of cuticle deposition during fruit development and involving substantial accumulation during ripening/overripening.

The fruit of the tropical species, mango (*Mangifera indica* L.) belongs to the order sapindales in the family *Anacardiaceae*, also known as the “king of fruits”, is a climacteric, fleshy and large drupe that has considerable commercial value^1^. The mango fruit varies in size, shape, color, fiber content, flavor, and taste. The exocarp region develops into a leathery protective skin that is smooth, green and waxy, including lenticels originated from stomata. When mango ripe, the skin changes to a pale green or yellow marked with red, according to cultivars^2^,^3^. After harvest and under native climatic conditions, mango fruit ripen within 6 to 7 days, but reach an overripe stage and spoil within 15 days^4^. As is typical of many fleshy fruit species, in addition to a loss of tissue integrity and microbial infection, postharvest desiccation and consequent oversoftening are important determinants of postharvest fruit quality^5^.

The exocarp influences the outward appearance of fruit, as reflected in characteristics such as color, glossiness, texture, and uniformity, and it also plays an important role in shelf life^6^. The exocarp is also referred to as the “peel”, and typically comprises the epidermal cell layer, together with layers of associated collenchyma, and even parenchyma cells, depending on how the peel was physically removed^7^. An important constituent of the exocarp is the cuticle, a hydrophobic layer composed mostly of cutin and waxes, which is synthesized by epidermal cells and represents the outermost barrier between aerial plant organs and the environment^8^. Cuticles have many functions, such as limiting water loss and gas diffusion, and providing protection against insects, pathogens, and ultraviolet radiation^9^. Their composition and physical properties are also suggested to contribute to commercially important fruit traits and postharvest shelf life^5^. In this regard, an improved understanding of the molecular processes involved in cuticle formation and modification has considerable potential value in designing strategies to improve fruit quality.

To date, most studies of cuticle related genes have been carried out using the vegetative organs of the model plant Arabidopsis (*Arabidopsis thaliana*). However, tomato fruit have become a model system for the study of the cuticle biology in fleshy fruits^10^,^11^,^12^, while additional important information has been gained from equivalent analyses of other fruits, such as apple (*Malus domestica* B.)^13^ and sweet cherry (*Prunus avium* L.)^14^.

However, despite the agronomic and economic importance of mango fruit, little is known about the molecular mechanisms that underly the biosynthesis of its cuticle. Moreover, only a few studies have addressed the changes in mango fruit cuticle composition and morphology during development and storage^15^,^16^, or in response to various treatments^17^. The major obstacle to further progress in mango genetic research is the limited availability of genomic data. However, next generation DNA sequencing methods and bioinformatic pipelines are facilitating the generation of genomic and transcriptomic analytical tools and resources. The application of such tools to study cuticle-associated genes will be of great value in elucidating many aspects of cuticle biology that currently are not well understood^5^.

The objective of this current study was to gain insight into cuticle biogenesis identifying putative cuticle-associated genes during mango fruit ontogeny. To this end, we used RNA Sequencing (RNA-Seq) to profile the transcriptomes of ripe and overripe fruit mango peels and further analyzed the expression profile of putative cuticle-associated genes by real time quantitative reverse transcription PCR (qRT-PCR). Patterns of gene expression were then correlated with the rate of cuticle accumulation during the fruit ontogeny. We discuss the putative roles of specific genes in the biosynthesis and transport of cutin and waxes during the cuticle formation.

## Results and Discussion

### Cuticle deposition during mango fruit ontogeny

**Figure 1:**
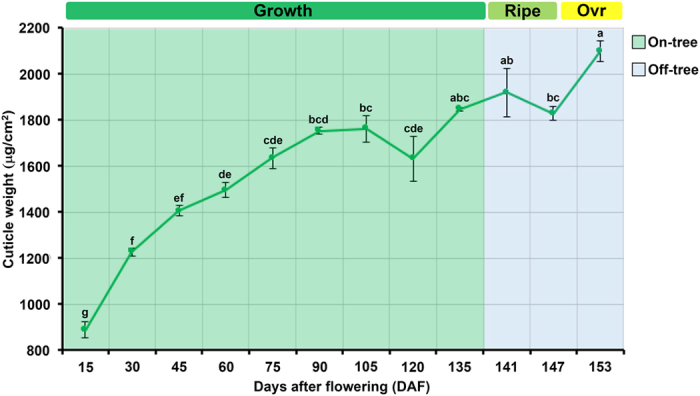
Cuticle accumulation. Cuticle deposition during mango fruit ontogeny. The X axis shows the stage of mango fruit development in days after flowering (DAF) and the Y axis shows the amount of cuticle. The error bars correspond to the standard error, as determined using three replicates samples, each consisting of 10 exocarp discs. Sampling points with different letters are significantly different based in variance analysis and Tukey test with 5% of significance. Ovr stands for overripe.

The weight of the fruit cuticle was 889 μg/cm^2^ at 15 days after flowering (DAF), and continued increasing during subsequent fruit development (30–105 DAF), reaching a maximum value of 1,763 μg/cm^2^, and then decreasing to 1,632 μg/cm^2^ at 120 DAF. After that, cuticular weight showed an increase of 1846 and 1920 μg/cm^2^ from 135 to 141 DAF, respectively. Unlike the foregoing pattern, a slight decrease to 1827 μg/cm^2^ was recorded at 147 DAF. Finally, the cuticle accumulation showed a large increase by the end of the storage time to reach 2100 μg/cm^2^ at 153 DAF, which is the maximum cuticular weight registered during mango fruit ontogeny (Fig. 1). This revealed a continuous pattern of cuticle deposition during fruit development and involving substantial accumulation during ripening/overripening. This is consistent with the cuticle accumulation pattern reported by Petit-Jiménez *et al*.^16^, for the same mango cultivar. A similar result was reported for orange fruit (*Citrus sinensis* Osbeck), where the weight of cuticle per unit increased by almost two-fold at 150 DAF compared with 120 DAF^18^. It has also been reported that apple fruit exhibit an increase in cuticular wax deposition during ripening, coincident with a burst in ethylene production^19^. Nonetheless, this pattern differs from that seen in many fruit species, where cuticle deposition ceases or decreases immediately after the phase of fruit expansion and before the initiation of the ripening^20^. For example, grape berry was reported to undergo a decrease in the amount of cuticular material after harvest^21^, and a similar phenomenon was seen in sweet cherry^14^. Moreover, in tomato, Yeats *et al*.^22^, reported that maximal cuticle accumulation occurred during the most rapid stage of fruit growth and thereafter, cuticle accumulation decreased until reaching the red ripe stage. However, to date nothing is known about its functional significance, or the factors that determine the various patterns of cuticular deposition^5^. The results of several studies suggest that cuticle biosynthesis depends on species and stage of development and it is influenced by genetics, physiological, environmental factors and postharvest handling^9^, which can explain the differences in the studies above mentioned.

**Figure 2:**
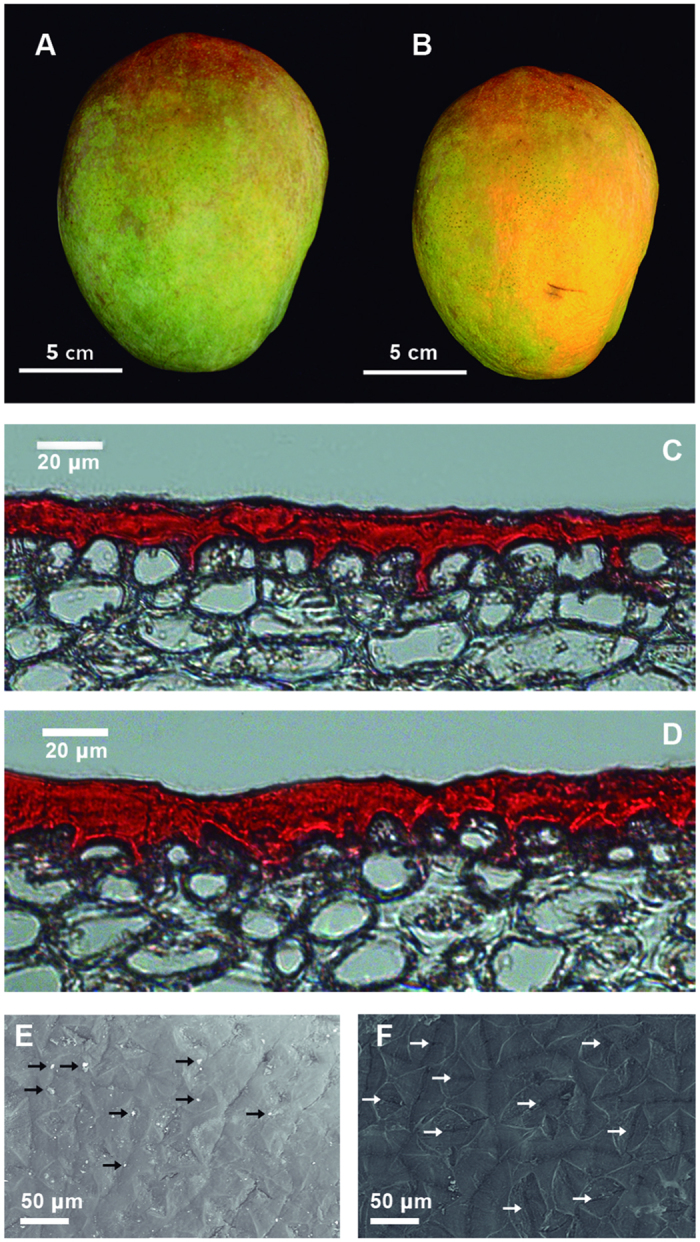
Fruit cuticle structural diversity. Mango fruit at the stages used for RNA-Seq analysis: ripe (**A**) and overripe (**B**). Cuticles stained with Oil Red from ripe (**C**) and overripe (**D**) mango fruit SEM images of cuticles from ripe (**E**;14.39 ± 0.37 µm) and overripe (F;16.64 ± 0.31 µm) stages. Arrows indicates scales (**E**) and holes (**F**), respectively.

There have been few studies of cuticles during fruit storage^20^. In this context, in order to identify potential changes in cuticle architecture during the ripe to overripe transition (Fig. 2A,B), we analyzed cuticle structural organization using light and SEM microscopy. Our results showed a slight increase in cuticle thickness and shape in overripe (16.64 ± 0.31 μm) (Fig. 2D) as compared with ripe stage fruits (14.39 ± 0.37 μm) (Fig. 2C), moreover, a student’s t-test confirmed significant differences in the cuticle thickness between both fruit developmental stages (p-value = 1.217E-05); which correlates with the differences in cuticle weight during the transition from ripe to overripe stages (Fig. 1). This finding agrees with previous reports showing that the cuticular wax density increased as the fruit develops, including during mature and overripe in mango cv. ‘Kensington Pride’^15^, ‘Keitt’^16^ and ‘Dashehari’^23^.

SEM imaging of the cuticles showed that the cuticular wax consisted of wax ridges, including a compact arrangement with few scales in ripe stage (Fig. 2E). Meanwhile, in overripe fruits we observed a smooth wax layer with no scales, and small holes due to cracks between the wax ridges and lenticels (Fig. 2F). Further, the development of cracks had been reported to occur as the fruit grew to maturity and ripened in mango^15^. Similar results were previously reported for ripe mango^24^, including loss of water and cell integrity.

### *De novo* assembly, functional annotation and classification of mango unigenes

**Figure 3:**
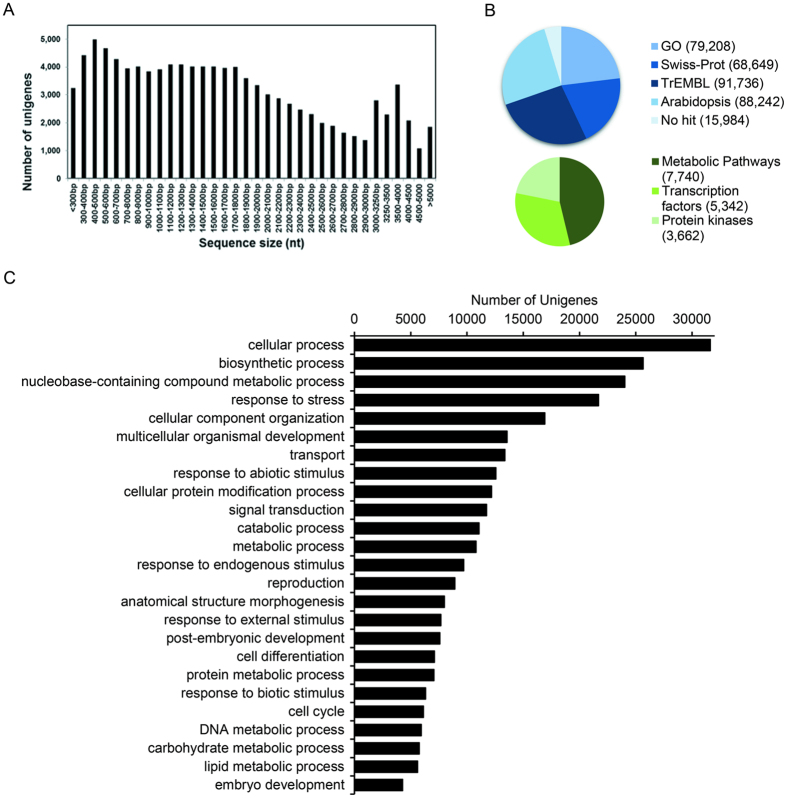
Mango RNA-Seq assembly annotation. (**A**) Length distribution of mango unigenes. (**B**) Summary of mango unigene annotation. (**C**) Top 25 GO terms in biological process category. The X axis indicates the number of unigenes for each biological process. The numbers indicate the annotated unigenes.

We sequenced six cDNA libraries (three ripe and three overripe) derived from mango (cv. ‘Keitt´) peels using an Illumina HiSeq™ 2500 operating in paired-end mode. This generated an average of approximately 74 million reads pairs from each ripe fruit peel library and 57 million read pairs from each overripe fruit peel library. After removing low-quality and adaptor sequences, a total of ~681 million reads (62.5 Gbp high-quality sequence) were used for *de novo* assembly. A total of 107,744 unigenes were assembled, with a total length of 184,977,733 bp, the robustness of this transcriptome data set is supported by the N50 value of 2,235 bp and mean length of 1,717 bp, which are among the highest values of previously mango transcriptomes reported (Supplementary Table S2). Unigenes were classified into four groups based on their length, 3,243 (3%) of the unigenes were <300 bp, 69,214 (64.23%) were 300–2,000 bp, 33,426 (31.02%) were 2,000- 5,000 bp, while 2,235 (2.07%) unigenes were longer than 5,000 bp (Fig. 3A). Our transcriptome analysis summary and the comparison with the previously analysis is shown in Supplementary Table S2.

The resulting sequence data were used to develop an online database called Mango RNA-Seq Database, which can be accessed at http://bioinfo.bti.cornell.edu/cgi-bin/mango/index.cgi. The annotation analysis using BLAST showed that the numbers of unigenes with significant hits (E-value ≤ 1E-5) to the Swiss-Prot, TrEMBL and Arabidopsis protein databases were 68,649 (63.7%), 91,736 (85.1%) and 88,242 (81.9%), respectively. A total of 15,984 unigenes had no significant matches in the Swiss-Prot and TrEMBL databases, and the remaining 91,760 unigenes were annotated using AHRD, among which 5,705 were annotated as unknown proteins (Fig. 3B and Supplementary Dataset S1).

A total of 79,208 (73.5%) unigenes were assigned into 9,945 Gene Ontology (GO) terms and, of these, 67,003, 68,347 and 67,160 were assigned at least one GO term in the ‘biological process’, ‘cellular component’ and ‘molecular function’ categories, respectively. The ‘biological process’ includes ‘cellular protein modification process’, ‘response to stress’, ‘carbohydrate metabolic process’, ‘lipid metabolic process’, ‘secondary metabolic process’, among others. The molecular functions were related to hydrolase activity, catalytic activity, transferase activity, kinase activity, lipid binding. Additionally, we identified the putative biochemical pathways represented in the assembled mango transcriptome. Our analysis showed that a total of 7,740 unigenes were annotated in 461 pathways, including triacylglycerol biosynthesis, phenylpropanoid biosynthesis, phospholipid biosynthesis II, cutin monomer biosynthesis and flavonoid biosynthesis, among others. Finally, we identified unigenes encoding transcription factors (TFs) and protein kinases. A total of 5,342 TFs were classified into 55 different families, of which the largest were MYB, C3H, NAC, bHLH and HB. MYB transcription factor have been reported as regulators of various plant responses, including in cuticle biosynthesis under drought stress^25^.

A total of 3,662 protein kinases were classified into 76 different families. In addition, the families with the largest number of unigenes were Domain of Unknown Function 26 (DUF26) Kinase, Receptor Like Cytoplasmic Kinase VII, SNF1 Related Protein Kinase (SnRK), GmPK6/AtMRK1 Family and LAMMER Kinase Family (Fig. 3B and Supplementary Dataset S1).

### Differential expression analysis

A comparative RNA-Seq analysis of peels from fruit at the overripe versus ripe developmental stages identified 1,616 and 3,733 unigenes that were up-regulated and down-regulated during overripening, respectively. An enrichment analyses of the up-regulated unigenes revealed that ~210 biological processes with associated GO terms, including those related to different ‘stress responses’, ‘lipid metabolic process’, ‘secondary metabolic process’, ‘cell wall metabolic process’ and ‘fruit ripening’. In the ‘molecular functions’ category (~125), the most represented GO terms assigned to unigenes were kynurenine-glyoxylate transaminase activity, cysteine-S-conjugate beta-lyase activity, long-chain-fatty-acyl-CoA reductase activity, fatty-acyl-CoA reductase (alcohol-forming) activity and acyl-[acyl-carrier-protein] desaturase activity (Supplementary Dataset S2).

For the down-regulated unigenes, the enrichment analysis identified ~550 significantly enriched biological processes, including those related to ‘cell wall polysaccharide metabolic process’, ‘homogalacturonan biosynthetic process’, ‘fruit development’, ‘response to ethylene’ and ‘secondary metabolic process’. The molecular functions (~185) down-regulated included, angiotensin receptor activity, inositol-3-phosphate synthase activity, choline dehydrogenase activity, serine transmembrane transporter activity and shikimate kinase activity (Supplementary Dataset S2).

**Figure 4:**
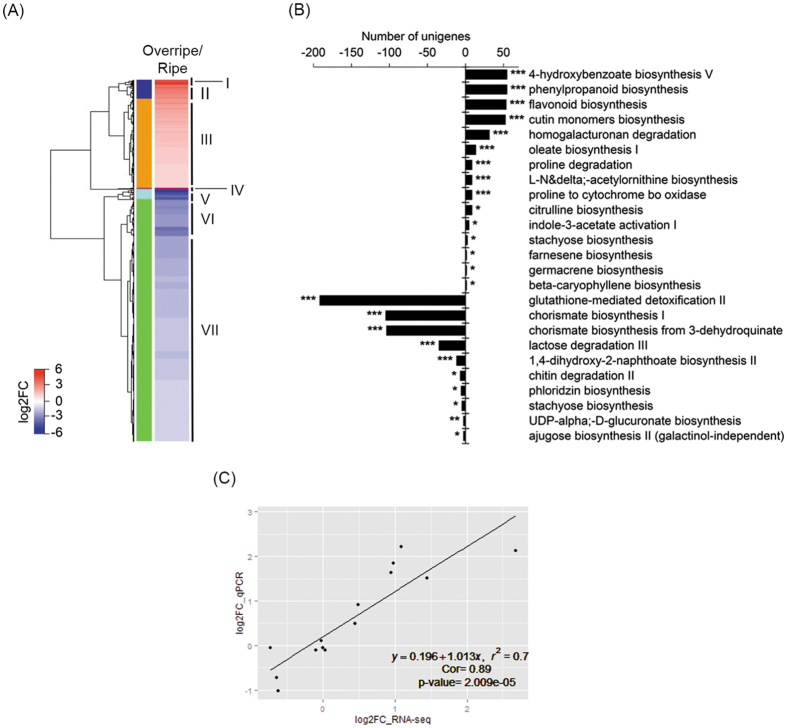
Differential expression analysis. (**A**) Heat-map of 5,349 differentially expressed unigenes. The Roman numeral in the right side and the left column colors indicate independent clusters and the color key indicates the log2 fold change of overripe versus ripe samples. (**B**) Graph showing the pathways enriched in the overripe versus ripe fruit peel comparison. Right and left side indicates the metabolic pathways enriched in the up-regulated and down-regulated unigenes during overripening, respectively. Asterisks indicate the statistical significance level, corrected p-value < 0.001 (***), corrected p-value < 0.01 (**), corrected p-value < 0.05 (*). (**C**) Graphing shows the Pearson Correlation Coefficient value between the gene expression ratios obtained from RNA-Seq and qRT-PCR analysis.

In addition, a clustering analysis of the 5,349 differentially expressed unigenes by expression levels identified 7 clusters (Fig. 4A). Clusters I and II comprised 66 and 213 induced unigenes during overripening, respectively, and included genes related to glutathione-mediated detoxification II, homogalacturonan degradation, fatty acyl-CoA reductase and starch biosynthesis. Interestingly for the goal of this study, cluster III, which contained 1,318 induced unigenes, included those with annotated functions related to cutin monomer biosynthesis, flavonoid biosynthesis, oleate biosynthesis and homogalacturonan degradation. Cluster IV included 19 strongly induced unigenes with no clear functional annotation. The down-regulated unigenes during overripening were grouped into 3 clusters (V, VI and VII), and included genes associated with cytochrome P450 activity, carbohydrate degradation, glutathione transferase, chalcone synthase, chorismate biosynthesis and ethylene biosynthesis (Fig. 4A and Supplementary Dataset S3).

A metabolic pathway enrichment analysis showed that the up-regulated unigenes encoded enzymes in 106 metabolic pathways, included: 4-hydroxybenzoate biosynthesis V, phenylpropanoid biosynthesis, cutin monomers biosynthesis, flavonoid biosynthesis, and homogalacturonan degradation. The down-regulated unigenes encoded enzymes in 131 pathways such as glutathione-mediated detoxification II, chorismate biosynthesis I, chorismate biosynthesis from 3-dehydroquinate, lactose degradation III and 1,4-dihydroxi-2-naphthoate biosynthesis II (Fig. 4B and Supplementary Dataset S4).

We also identified the differentially expressed unigenes encoding TFs and protein kinases. A total of 92 TFs in 20 families were up-regulated during overripening, including: HSF, MYB and bHLH. Conversely, 342 TFs in 25 families were down-regulated during overripening, including NAC, C2C2-GATA and G2-like. We also observed that 54 protein kinases in 19 families were up-regulated during overripening, including Leucine Rich Repeat Kinase XI & XII, Putative receptor like protein kinase and CRPK1 Like Kinase (Types 1 and 2). Conversely, 119 proteins kinases in 18 families were down-regulated during overripening, including SNF1 Related Protein Kinase (SnRK), CTR1/EDR1 Kinase and Ankyrin Repeat Domain Kinase (Supplementary Tables S3,S4).

In order to corroborate the expression levels of the unigenes assembled by RNA-seq analysis, we performed a qRT-PCR expression analysis. For this, we selected several unigenes based on homology to genes associated with known roles in biosynthesis, regulation and transport of cuticle for other plants (Table 1). We used the same RNA samples from ripe and overripe mango peels used for RNA-Seq analysis to synthesize cDNA. We compared the log2fold change (overripe versus ripe) obtained by RNA-Seq and qRT-PCR analyses. Linear regression analysis showed a *r*^*2*^ value of 0.798 and a Pearson correlation coefficient of 0.893, with a p-value of 2.009E-5, indicating a close correlation between transcript abundance quantified by qRT-PCR and the transcription profile obtained by RNA-Seq data, thereby supporting the accuracy of the data (Fig. 4C).

### Cuticle biosynthesis

The biosynthesis of waxes, cutin, isoprenoids and phenylpropanoids involve four major metabolic pathways that are involved in tomato cuticle formation^6^. Lipid metabolism is central to cuticle assembly as it supplies the precursors for the biosynthesis of wax and cutin. Indeed, Suh *et al*.^26^, reported that over half of the fatty acids synthesized in the Arabidopsis stem epidermis are exported into the cuticle. In addition, Mintz-Oron *et al*.^7^, reported that the expression of 15% of the genes associated with fatty acid metabolism, wax and cutin was up-regulated in the tomato peel during cuticle formation, while secondary metabolites synthesized via the phenylpropanoid pathway are often constituents of cuticular waxes. In agreement with these data, our transcriptome analysis revealed a total of 136 and 45 unigenes, as associated with ‘lipid metabolism process’ (GO:0006629) and ‘cutin biosynthetic process’ (GO:0010143), respectively, whose expression was up-regulated during overripening. Additionally, the molecular functions most represented were ‘long-chain-fatty-acyl-CoA reductase activity (GO:0050062)’ and ‘fatty-acyl-CoA reductase (alcohol-forming) activity (GO:0080019)’, with 45 unigenes each. We identified 53 and 4 unigenes, involved in the cutin biosynthesis pathway (PWY-321), that were up-regulated or down-regulated, respectively. In addition, we identified 123 and 110 unigenes whose expression was up-regulated involved in ‘secondary metabolic process’ (GO:0019748) and ‘phenylpropanoid metabolic process’ (GO:0009698), respectively, suggesting that the pathways related to the biosynthesis of cuticle components were very active in the peel during mango fruit overripening.

### Gene expression during cuticle biosynthesis

**Figure 5:**
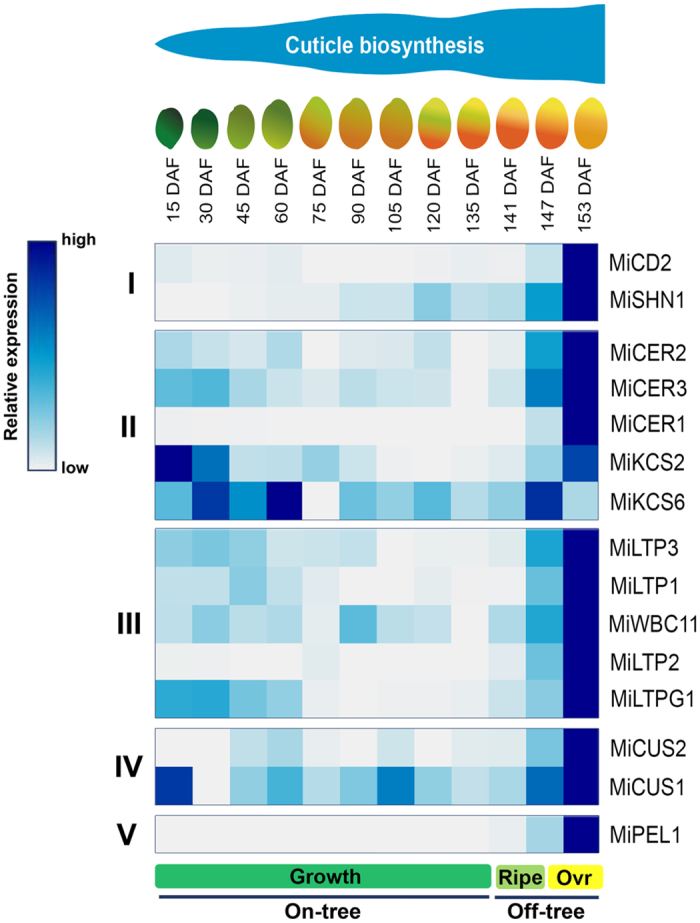
Heat map of expression profile of putative cuticle-associated genes and cuticle accumulation during mango fruit ontogeny. Time course of the expression of selected genes during fruit growth and ripening. The genes were classified into five groups according the role in cuticle biosynthesis. I: Regulation; II: Wax biosynthesis; III: Wax transport; IV: Cuticle assembly and V: Control for ripening. The cuticle deposition is indicated above the fruit development stages. Ovr stands for overripe.

In order to gain further insight into mango cuticle biogenesis, we analyzed the expression of fifteen putative cuticle-associated genes during fruit ontogeny (Fig. 5).

### Regulation

The regulation of cuticle biosynthesis involves feedback from the cuticle components and with interacting metabolic pathways playing a role in responses to pathogen and environmental stress^8^. In Arabidopsis, the first cuticle-associated transcription factor, the AP2-domain super family member SHINE1/WAX INDUCER1 (AtSHN1/WIN1) induces the expression of a large numbers of gene encoding enzymes that are involved in fatty acid elongation and the formation of aliphatic compounds^27^,^28^. We identified a mango homolog of *AtWIN1/SHN1, MiWIN1/SHN1* (MIN047952), encoding a protein with 68% identity to AtWIN1/SHN1. The gene showed weak expression between 90 and 141 DAF, but this expression increased substantially during the storage period (147–153 DAF), coincident with cuticle accumulation. This is also consistent with the observation that overexpressing the tomato homolog, *SlSHN1*, induced a higher cuticular wax deposition compared with isogenic tomatoes lines^29^. Additionally, in sweet cherry (*Prunus avium*), the *PaWIN/SHN1* gene showed high expression levels at 21 days after full bloom, again during the major phase of cuticle accumulation^14^.

The transcription factor, *SlCD2 (Solanum lycopersicum CUTIN DEFICIENT 2*), a member of the HD-ZIP IV family, was identified as a regulator of cutin biosynthesis in tomato fruit^10^. Accordingly, fruit of the *cd2* mutant had an extremely thin cuticle and increased susceptibility to microbial infection. In addition, Matas *et al*.^11^, reported that the *CD2* was the most differentially expressed transcription factor in the outer epidermis than in vascular tissues in tomato fruit. In this current study, we identified a mango homolog, *MiCD2* (MIN074277), encoding a protein with 86% identity to the tomato *SlCD2*. This gene showed low expression during the early and intermediate stages of development (15–141 DAF), but its transcript levels increased strongly during storage (147–153 DAF), coincident with cuticle accumulation, supporting its role in cuticle formation.

### Wax biosynthesis

The *CER1* gene encodes an aldehyde decarbonylase enzyme and catalyzes the conversion of long chain aldehydes to alkanes, a key step in wax biosynthesis^30^,^31^. Bourdenx *et al*.^32^ demonstrated that the overexpression of *CER1* specifically increased the levels of odd-carbon-numbered alkanes; mainly C27, C29, C31, and C33 alkanes. We identified a mango homolog of *AtCER1, MiCER1* (MIN107433), encoding a protein with 64% sequence identity. This gene showed very low expression during early and intermediate stages of development (15–141 DAF), and a strong increase in expression during ripening and storage. Albert *et al*.^13^ observed a correlation between the abundance of alkanes in apple fruit with the expression of *MdCER1* gene. Moreover, Broun *et al*.^28^, reported that *AtCER1* expression was induced by overexpression of *AtWIN1/SHN1*. We detected the presence of *MiSHN1* transcripts prior to the expression increase of *MiCER1*, which is consistent with an analogous regulatory system in mango, although this need to be tested experimentally.

The *CER2* gene encodes a putative BAHD acyltransferase involved in the elongation of alkanes beyond C28. In Arabidopsis, the *cer2* mutant lacks waxes longer than C28, suggest that *CER2* plays a role in very long chain fatty acid (VLCFA) synthesis^33^,^34^. We identified a mango homolog of *AtCER2, MiCER2* (MIN052433) with a low expression level during early and intermediate stages of fruit development (15–141 DAF) and increased expression during ripening and storage; thus *MiCER2* expression correlated with cuticle accumulation.

*CER3 (WAX2*) was suggested to form an enzyme complex catalyzing the conversion of very long chain (VLC) acyl-CoAs to VLC alkanes^31^. The total wax amount on *Atcer3* mutant leaves and stems was reported to be reduced by 78%, and a reduction in the amount of aldehydes, alkanes and secondary alcohols was observed^35^. We identified a mango homolog of *AtCER3, MiCER3* (MIN064126) with low expression during early and intermediate stages of development (15–141 DAF), and high expression during ripening and storage.

The *KCS2/Daysi* gene encodes a 3-Ketoacyl-CoA synthase 2 and it is functionally redundant in cuticular wax and root suberin biosynthesis catalyzing the two-carbon elongation leading to C22 VLCFA^36^. We identified a mango homolog of *AtKCS2, MiKCS2* (MIN101804) that was expressed at high levels during the early stages of fruit development (15–30 DAF). After that, expression was reduced during intermediate stages of development (75–141 DAF), but increased again during ripening and storage, although not reaching the levels observed during early stages. The expression of *MiKCS2* did not correlate with cuticle accumulation, and its function may be more related with changes in cuticle composition. This idea awaits to be experimentally tested.

The *KCS6 (CER6* or *CUT1*) gene encodes a 3-ketoacyl-CoA synthase 6^37^. A loss-of-function tomato mutant showed a reduction in *n*-alkanes and aldehydes with chain lengths beyond C30 in waxes of leaf and fruit^38^. We identified a mango homolog of *AtKCS6, MiKCS6* (MIN040156), with high expression during early fruit development stages (15–45 DAF), reaching the maximum expression at 60 DAF. After that, *MiKCS6* showed low expression during intermediate stages of development (75–141 DAF) and expression levels similar to the initial stages during ripening and storage. Thus, *MiKCS6* expression did not correlate with cuticle accumulation. A similar pattern was observed by Alkio *et al*.^14^, in sweet cherry, where *PaKCS6* expression did not show an increase in expression during cuticle deposition. Conversely, Yeats *et al*.^22^, reported high expression of a tomato homolog, *SlKCS6 (CER6*) gene during the most rapid phase of fruit expansion, corresponding to a phase of substantial cuticle accumulation. The expression pattern of *SlKCS6* is therefore different developmentally compared with the expression of *MiKCS6*; however, both genes show high expression levels during the initial stages of fruit development.

### Wax transport

Cuticle biosynthesis requires an extensive transport of lipids through the plasma membrane and cell wall of the epidermal cells. ATP binding cassette (ABC) transporters located in the plasma membrane of epidermal cells are required for both cutin and wax deposition^39^. In Arabidopsis, the ABC plasma membrane transporter, *AtWBC11*, is involved in the export of both cutin precursors and wax during cuticle development^40^. We identified a mango homolog of *AtWBC11, MiWBC11* (MIN106958), with low expression during early and intermediate stages of fruit development (15–141 DAF), and high expression during ripening and storage, which correlated with cuticle deposition. Supporting these results, Bird *et al*.^40^, reported that alkane levels of mutant *Atwbc11* correlated with the levels of detectable *AtWBC11* transcript and Alkio *et al*.^14^, reported a correlation in sweet cherry, between transcript levels of *PaWBC11* and the rate of cuticle deposition at earlier stages of fruit development.

The mechanism of structural lipid transport through the cell wall is not well understood, although lipid transfer proteins (LTPs), which are abundantly expressed in the epidermis and are secreted into the apoplast, have been proposed to be involved^41^. Yeats *et al*.^22^, reported changes in the expression of LTPs during tomato fruit ontogeny coincident with cuticle biosynthesis. We identified three mango homologs of tomato LTP SGN-U579687: *MiLTP1* (MIN026365), *MiLTP2* (MIN018326) and *MiLTP3* (MIN107167). Our expression analysis showed that *MiLTP1* and *MiLTP2* were expressed at low levels during early (15–141) and intermediate stages (15–135 DAF) of fruit development. In contrast, *MiLTP3* showed moderate expression during early development (15–90 DAF) and low expression at the intermediate stage (105–141 DAF). However, all the three genes showed high expression during ripening and storage. These results suggest that these genes could be participating in the cuticle accumulation during the late stages. Mintz-Oron *et al*.^7^ also reported high expression of SGN-U579687 in tomato fruit peels and we noted a higher expression of *MiLTP1* compared with all other genes analyzed.

In Arabidopsis, the export of some wax compounds appears to be facilitated by glycosylphosphatidylinositol (GPI)-anchored lipid-transfer (LTPG) proteins. LTPG1 protein was localized in the plasma membrane and an *Atltpg1* mutant with decreased expression showed a reduced wax load, mainly in C29 alkanes, and higher susceptibility to infection by *Alternaria brassicicola*, than the wild type^42^,^43^. We identified a mango homolog of *AtLTPG1, MiLTPG1* (MIN012243) that showed moderate expression during early stages of fruit development (15–60 DAF), low expression in intermediates stages (75–135 DAF) and maximal expression during ripening and storage which suggest a possible role for this gene in the export of cuticle wax components in mango fruit. In sweet cherry, Alkio *et al*.^14^, reported positive correlations between *PaLTPG1* expression and cuticle accumulation during early fruit development but expression did not correlate in later development.

### Cuticle assembly

The GDSL-motif lipase/hydrolase enzyme, *SlCUS1* (originally termed *CD1*), is a cutin synthase that catalyzes the polymerization of cutin monomers in the growing cuticle of tomato fruit^44^. We identify a mango homolog of *SlCUS1, MiCUS1* (MIN010966) that showed high expression during early fruit development (15 DAF), moderate expression during intermediate stages (45–141 DAF) and high expression during ripening and storage. Yeats *et al*.^19^, reported that *SlCUS1* was maximally expressed during the most rapid phase of fruit expansion and cuticle accumulation, but not during ripening, which is consistent with the pattern of cuticle deposition late in mango fruit development, but not in tomato.

Another GDSL-motif lipase/hydrolase (SGN-U583101) gene was reported to show a high expression in the epidermis^22^. We identified a mango homolog of SGN-U583101, which we named *MiCUS2* (MIN031338). This gene showed a low gene expression during initial and intermediate stages, increasing expression at 147 DAF and maximum expression at 153 DAF. This data correlated with cuticle deposition during ripe and overripe stage. Conversely, Yeats *et al*.^22^, reported that SGN-U583101 was maximally expressed at 5 DPA which corresponds with a very early stage of fruit development.

As a control for ripening related gene expression, we also analyzed the expression of *MiPEL1* (AY987389), which encodes a pectate lyase in mango fruit. *MiPEL1* expression increased only at the ripe and overripe stage, in agreement with previous report where it had been associated with mango softening^45^.

Our transcriptome data derived from mango peel represents a valuable resource for future studies of mango fruit biology, but more specifically we have identified large numbers of unigenes, involved in several metabolic processes, related to mango fruit development, such as the synthesis or degradation of lipids, cutin, secondary metabolites and cell wall polysaccharides, among others.

Interestingly, the functional RNA-Seq analysis indicates that the cutin monomers biosynthesis pathway is enriched during overripening, which correlates with the microscopic and gravimetric analyses of cuticle accumulation. This revealed a complex continuous pattern of cuticle deposition during fruit development, involving substantial accumulation during ripening/overripening. The identification and analysis of the putative cuticle-associated genes expression provides the first insight to understand cuticle biosynthesis in mango fruit and indicate a close correlation with cuticle accumulation. However, further studies are required to confirm the role of these genes during fruit development. The cuticle-associated genes identified in this study will help in the elucidation of the molecular mechanism underlying cuticle biosynthesis and in the design of strategies to increase the postharvest shelf life of mango fruits.

## Material and Methods

### Plant materials

Mango (*Mangifera indica L*., cultivar ‘Keitt’) fruit used for RNA-Seq profiling were obtained from a commercial store at Ithaca, New York, USA. The fruit were divided into two stages: ripe and overripe (storing them at room temperature approximately 20 °C and 60–65% relative humidity for 12 days). Mango fruit of the same cultivar used for qRT-PCR analysis were hand harvested every 15 days after flowering (DAF) until ripening from trees in a commercial orchard and a packinghouse located at El Porvenir, Ahome, Sinaloa, Mexico (www.agricoladaniella.com.mx). After ripening, the fruit were stored at 20 °C and 60–65% relative humidity for 18 days, with sampling carried out every six days.

### RNA-Seq library construction and sequencing

Peel samples from three fruit were pooled to create a single biological replicate and three independent biological replicates of both ripe and overripe fruit were analyzed. Total RNA was extracted as previously described^46^. Strand-specific RNA-Seq libraries were constructed using the protocol described in Zhong *et al*.^47^. The resulting six RNA-Seq libraries were sequenced on an Illumina HiSeq 2500 system (Illumina Inc. San Diego, CA, USA) in paired-end mode with a read length of 100 bp in the Institute of Biotechnology at Cornell University (http://www.biotech.cornell.edu/brc/genomics-facility). The raw sequencing reads were deposited in the NCBI Sequence Read Archive (SRA) under the accession number SRP043494.

### RNA-Seq data processing, *de novo* assembly and annotation

RNA-Seq reads were first processed to trim adapter and low quality sequences using Trimmomatic^48^. Reads shorter than 40 bp were discarded. The resulting high-quality cleaned reads were assembled *de novo* into contigs using Trinity, with “min_kmer_cov” set to 10^49^. Following assembly, the high-quality cleaned reads were aligned to assembled contigs using Bowtie^50^, allowing 3 mismatches. Following alignments, expression values (FPKM; fragments per kilobase of exon model per million mapped reads) were derived for each contig. Lowly expressed contigs (FPKM < 0.002), and contigs with a low ratio number of sense to antisense reads (<0.1) were discarded, as those sense reads may have been derived from incomplete digestion of the second strand during the strand-specific RNA-Seq library construction.

The resulting assembled contigs were then used to query the GenBank Nucleotide (nt) database (http://www.ncbi.nlm.nih.gov/genbank/) using the Basic Local Alignment Search Tool (BLAST)^51^ function, and those having hits only to sequences from viruses, bacteria, and archaea were discarded. Next, rRNA, low-complexity, and polyA/T sequences were removed or trimmed from the contigs using SeqClean (https://sourceforge.net/projects/seqclean/). To remove redundancies in the contigs, the remaining contigs were further *de novo* assembled using iAssembler^52^ with 97% minimum percent identify.

The final assembled mango unigenes were used to query the UniProt (Swiss-Prot and TrEMBL; http://www.uniprot.org/) and Arabidopsis protein databases (https://www.arabidopsis.org/) using BLAST, with a cutoff E-value of 1E-5. Based on the BLAST results, functional descriptions were assigned to each mango transcript using automated assignment of human readable descriptions (AHRD: https://github.com/groupschoof/AHRD). Gene ontology (GO) terms were assigned to the mango assembled transcripts based on the GO terms annotated to their corresponding homologs in the UniProt database^53^. Biochemical pathways were predicted from the mango transcripts using the Pathway Tools program^54^. Transcription factors and protein kinases were identified and classified into different families using the iTAK pipeline (http://bioinfo.bti.cornell.edu/tool/itak).

### Gene expression quantification and differential expression analysis

The high-quality cleaned RNA-Seq reads were aligned to the assembled mango transcripts using Bowtie^50^, allowing 3 mismatches and only keeping the best alignments. Following alignments, raw counts for each mango transcript, and in each sample, were derived and normalized to FPKM. Genes that were differentially expressed (fold changes ≥2.0 and adjusted p-value < 0.05) between overripe and ripe fruits were identified with the DESeq package^55^. GO terms enriched in the set of differentially expressed genes (adjusted p value < 0.05) and altered pathways were identified using Plant MetGenMAP^56^.

### cDNA synthesis and real-time quantitative reverse transcription PCR for the developmental time course

DNA sequences of the mango homologs of tomato and Arabidopsis genes associated with cuticle biosynthesis, transport and regulation were obtained from our mango database using BLASTX (E-value ≤ 1E-5). The Coding DNA Sequence (CDS) and deduced amino acid sequence were obtained using Open Reading Frame Finder (http://www.ncbi.nlm.nih.gov/gorf/gorf.html). Signal-3L was used to identify signal peptides^57^ and PredGPI predictor to identify glycosylphosphatidylinositol (GPI) domains^58^. Oligonucleotide primers were designed according to Thornton and Basu^59^ and are listed in Supplementary Table S1.

Peels were pooled to generate replicates and RNA extracted as above. A total of 2 μg of total DNase-treated RNA was used for first strand cDNA synthesis using SuperScript II reverse transcriptase and oligo(dT) primers (Invitrogen), according to the manufacturer’s instructions.

Quantitative PCR analyses were performed using a StepOne™ Real-Time PCR System (Applied Biosystems, Foster City, CA, USA). The cDNA samples were diluted 5-fold with water and 1 μl was used as a template for each 20 μl quantitative PCR, prepared using HotStart-IT SYBR Green qPCR Master Mix (2X) (Affymetrix, Santa Clara, CA, USA) in biological triplicates. The thermal cycling conditions consisted of 2 min at 95 °C, followed by 40 cycles at 95 °C for 15 s and 60 °C for 1 min. The specificity of the PCR products was determined by high-resolution melt curve analysis, gel electrophoresis and by sequencing, carried out at Macrogen (Macrogen Inc., Seoul, South Korea).

Statistical analysis of the relative expression results was carried out using the relative expression method^60^, with MiActin1 (GenBank accession No. JF737036) as a reference gene^61^, assuming a PCR efficiency of 1.0 for all genes. For each gene, expression was linearly normalized, with a value of 0.0 assigned to the stage with the lowest expression and 1.0 to the stage with the highest expression. Normalized gene expression profile data were used to create a heat map using heatmap.2 in the gplots package in R library (http://cran.r-project.org/web/packages/gplots/index.html).

### Microscopy

Peels from both ripe and overripe fruits was harvested, fixed, cryoprotected, and embedded as in Buda *et al*.^62^. Cryosections of each sample were transferred at room temperature onto VistaVision HistoBond slides (VWR Radnor, PA, USA), dried and stained with Oil Red O saturated in 60% isopropanol (Alfa Aesar, Ward Hill, MA, USA). Sections were imaged with a Zeiss AxioImager A1 microscope equipped with a Zeiss EC-Plan NeoFluar 3100/1.3 oil immersion objective, a Zeiss AxioCam MRc color video camera, and Zeiss AXIOVs40 4.6.3.0 software. Images were obtained using DIC optics on an AxioImager A1 microscope equipped with an EC-Plan NeoFluar 40x/0.75 objective and an AxioCam Mrc color video camera (Zeiss, Oberkochen, Germany). Cuticle thickness measurements were made on digitized micrograph images using ImageJ software (https://imagej.nih.gov/ij/). Ten replicates from several fruits were used for ripe and overripe stage, respectively. Each replicate consisted of 5 measurements made on five sections from the same sample. For SEM, the preparation technique is described next: 2 cm × 2 cm squares of surface tissue were quickly excised with a razor blade and adhered to small drop of low temperature glue on a 3 cm by 3 cm nitrocellulose sheet. The platform was rapidly plunged into liquid nitrogen and cooled for 30 sec. The platform was then secured to a liquid nitrogen-cooled JEOL cryostage (JEOL; www. jeolusa.com) and then placed into a JEOL 6480 Variable Pressure Scanning Electron Microscope. Specimens were imaged at 20 kV, 30 Pa (Pascal) pressure and spot size of 60.

### Isolation and quantification of the fruit cuticle

Three replicate samples, each consisting of 10 exocarp discs, were collected for each developmental stage. The cuticles and gravimetric analysis during mango ontogeny were carried out as previously described^16^. Data was analyzed by using variance analysis based in a complete randomized design with a significance level of 5%. Tukey test was run when significant differences were found.

## Additional Information

**How to cite this article**: Tafolla-Arellano, J. C. *et al*. Transcriptome Analysis of Mango (*Mangifera indica* L.) Fruit Epidermal Peel to Identify Putative Cuticle-Associated Genes. *Sci. Rep.* **7**, 46163; doi: 10.1038/srep46163 (2017).

**Publisher's note:** Springer Nature remains neutral with regard to jurisdictional claims in published maps and institutional affiliations.

## Supplementary Material

Supplementary Dataset S1

Supplementary Dataset S2

Supplementary Dataset S3

Supplementary Dataset S4

Supplementary Table S1-S2

Supplementary Table S3-S4

## Figures and Tables

**Table 1:** Characterization of cuticle-associated genes in mango.

Gene ID	Mi ID	Best hit ID (E value)	Protein identity/Query cover (%)	Gene product	Role	References
MiSHN1	MIN047952	AT1G15360.1 (1E-78)	63/100	AP2/EREBP-type transcription factor SHINE 1	Regulation	25,26
MiCD2	MIN074277	Solyc01g091630.2 (0.0)	82/100	Homeobox-leucine zipper protein ATHB-9	Regulation	8,9
MiCER1	MIN107433	AT1G02205.2 (0.0)	63/98	Aldehyde decarbonylase	Wax biosynthesis	28, 29, 30
MiCER2	MIN052433	AT4G24510.1 (1E-98)	41/96	BAHD acyltransferase	Wax biosynthesis	31,32
MiCER3	MIN064126	AT5G57800.1 (0.0)	69/98	Fatty acid reductase	Wax biosynthesis	29,33
MiKCS2	MIN101804	AT1G04220.1 (0.0)	79/97	3-Ketoacyl-COA Synthase 2	Wax biosynthesis	34
MiKCS6	MIN040156	AT1G68530.1 (0.0)	85/100	3-Ketoacyl -COA Synthase 6	Wax biosynthesis	35,36
MiWBC11	MIN106958	AT1G17840.1 (0.0)	86/98	ABC Transporter White-Brown Complex Homolog Protein 11	Wax Transport	38
MiLTP1	MIN026365	SGN-U579687 (1E-36)	54/97	Lipid transfer protein	Wax Transport	19
MiLTP2	MIN018326	SGN-U579687 (6E-29)	48/96	Lipid transfer protein	Wax Transport	19
MiLTP3	MIN107167	SGN-U579687 (1E-16)	37/90	Lipid transfer protein	Wax Transport	19
MiLTPG1	MIN012243	AT1G27950.1 (2E-53)	55/76	Lipid transfer protein	Wax Transport	40,41
MiCUS1	MIN010966	SGN-U585129 (0.0)	74/97	Cutin synthase	Cuticle assembly	19,42
MiCUS2	MIN031338	SGN-U583101 (3E-90)	42/90	Cutin synthase	Cuticle assembly	19
MiPEL1	MIN009006	AY987389 (0.0)	99/100	Pectate lyase	Control for ripening	43

Mango genes analyzed in this study were named after the most similar Tomato and Arabidopsis genes and including a prefix “Mi” for *Mangifera indica*.
