# Supplementary material for: Transcriptome Analysis of Mango (Mangifera indica L.) Fruit Epidermal Peel to Identify Putative Cuticle-Associated Genes
Source: Sci Rep. 2017 Apr 20;7:46163. doi: 10.1038/srep46163 (PMC5397871; doi:10.1038/srep46163)
Supplement: Supplementary Table S1-S2 [file srep46163-s5.pdf]

Transcriptome Analysis of Mango (*Mangifera indica* L) Fruit Epidermal Peels Provides Insights into Cuticle Biosynthesis

Julio C. Tafolla-Arellano, Yi Zheng, Honghe Sun, Chen Jiao, Eliel Ruiz-May, Miguel Hernández-Oñate, Alberto González-León, Reginaldo Báez-Sañudo, Zhangjun Fei, Jocelyn K.C. Rose, Martín E. Tiznado-Hernández.

Supplementary Table S1. DNA sequence of the primers used for the analysis of gene expresion by real time quantitative reverse transcription PCR.

Gene ID	Forward primer 5´→3´sequence	Reverse primer 5´→3´ sequence	Amplicon lenght from cDNA (bp)
**MiSHN1**	GGCTCTTGGGTCTCTGAG	CCTCTTCAGCCGTCTCAA	79
**MiCUS1**	GACAAGGACCCTACAATGGAATTGG	GATCTGTTGTACGATAACTCTGCCG	131
**MiCD2**	TAATGGACCCACAAACGGAAACAAT	TAGCTGTAGGAAGACTGTTCACCAA	110
**MiCUS2**	TCTACTGGACAGTCCTTGTGTTTCA	AGTTGTTGTTTCCCACATCAACCAA	111
**MiCER1**	GATTGTTTCTACCACTTAACACC	CACCCTTCTTGGAAGCCAATTC	90
**MiCER2**	GGAGGAAGAAAGTGAAAG	TTCAACCCATAAACATCCG	90
**MiCER3**	GAGGAGCCAAGAATTGAAT	GCATGTTGCTGTAGGAGTT	97
**MiKCS2**	GAATCTGGAGCTGAGTGA	CGATCACCTCTCTTTATCCT	135
**MiKCS6**	TCTTCTTCGTCCCTCTGGTA	CGTCAACTGGTGTCTTGA	154
**MiWBC11**	GAGATAGAGACGAGCAAG	CTCCCACAAGTTCTGTATTAG	106
**MiLTP1**	CATCCATCTCAGGCATCAACTA	ATGGGCTGATCTTGTAAGGG	84
**MiLTP2**	GGCATTCATAGCTGTGCT	GGTCACTTGTTCGCATGTTAT	82
**MiLTP3**	TGCAAAATGCAGCTAAAGGA	GTTGGTGGAGGTGCTGATCT	107
**MiLTPG1**	CTTCCCACTGCCTGTCAAAT	GAAGATAGCCGCATCTGGAG	93
**MiPEL1**	ATGGCGGTTTCTCCTAGA	TCACTGTCGATGCTTTAACG	85
**MiActin1**	CGTTCTGTCCCTCTATGCCA	AGATCACGGCCAGCAAGATC	141

Supplementary Table 2. Summary of mango transcriptomes studies. We compared our transcriptome analysis with previous studies directed to different tissues.

	**In this study**	**Azim *et al.*, 2014**	**Wu *et al.*, 2014**	**Luria *et al.*, 2014**	**Dautt-Castro *et al.*, 2015**
**Tissue Analyzed**	Peel	Leaf	Pericarp and pulp	Peel	Mesocarp
**Mango Cultivar**	Keitt	Langra	Zill	Shelly	Kent
**Total sequenced bases**	62.5 Gb	>1 Gb	6.1 Gb	8.6 GB	4.8 Gb
**Total unigenes**	107,744	30,509	54,207	57,544	52,948
**Total assembled bases**	184,977,733	16,354,267	-	-	-
**Average length unigenes**	1,717 bp	536	838	863.3	836
**Largest unigenes**	12,271 bp	-	-	-	8,713
**N50**	2,235 bp	687	1,328	1,598	1456
**Sequencing system**	HiSeq 2500	HiSeq 2000	HiSeq 2000	HiSeq 2000	Genome Analyzer GAIIx II
**Sequence Read Archive Accession Number**	SRP043494	SRR947746	SRP035450	SRX375390	SRP045880
**Swiss-Prot**	68,649 (63.7%)	14,447 (47.5%)	26,380 (48.67%)	-	25,154
**TrEMBL**	91,736 (85.1%)	-	-	-	-
**Arabidopsis Database**	88,242 (81.9%)	-	-	-	-
**NCBI non-redundant (NR)**	-	24,593 (80 %)	42,515 (78.43%)	35,719 (62.07%)	32,560
**GO terms**	79,208 (73.51%)	21,054 (69%)	35,198 (64.93%)	28,317 (49.2%)	29,844
**Unigenes/Pathways**	17,686/461 Pathway Tools	13,561/293 KEGG pathways	23,741/128 KEGG pathways	-	7,458/327 KEGG pathways
**Transcription Factor/Families**	5,432/55	-	-	-	-
**Protein kinases/Families**	3,662/76	-	-	-	-

References

Azim, M.K. Khan, I. Zhang, Y. Characterization of mango (*Mangifera indica L.*) transcriptome and chloroplast genome. Plant Mol Biol. **85,**193-208;10.1007/s11103-014-0179-8 (2014).

Dautt-Castro, M. *et al*. Mango (*Mangifera indica L.)* cv. Kent fruit mesocarp *de novo* transcriptome assembly identifies gene families important for ripening. Front Plant Sci. **6,** 62;10.3389/fpls.2015.00062 (2015).

Luria, N. *et al*. *De-novo* assembly of mango fruit peel transcriptome reveals mechanisms of mango response to hot water treatment. BMC Genomics **15,** 957; 10.1186/1471-2164-15-957 (2014).

Wu, H-x. *et al*. Transcriptome and proteomic analysis of mango (*Mangifera indica Linn*) fruits. Journal of Proteomics **105,** 19-30. J Proteomics;10.1016/j.jprot.2014.03.030 (2014).
